# Perceptions of postnatal depression and health care needs in a South African sample: the “mental” in maternal health care

**DOI:** 10.1186/s12905-014-0140-7

**Published:** 2014-11-12

**Authors:** Tasneem Kathree, One M Selohilwe, Arvin Bhana, Inge Petersen

**Affiliations:** Psychology, School of Applied Human Sciences, University of KwaZulu-Natal, Mazisi Kunene Road, Durban, 4041 South Africa

**Keywords:** Maternal, Postnatal, Mental health, South Africa, Task-sharing, Low income

## Abstract

**Background:**

Maternal mental health care is a neglected area in low and middle income countries (LAMIC) such as South Africa, where maternal and child health care priorities are focused on reducing maternal and infant mortality and promoting infant physical health. In the context of a paucity of mental health specialists, the aim of this study was to understand the explanatory models of illness held by women with maternal depression with the view to informing the development of an appropriate counselling intervention using a task sharing approach.

**Methods:**

Twenty semi-structured qualitative interviews were conducted with mothers from a poor socio-economic area who were diagnosed with depression at the time of attending a primary health care facility. Follow-up interviews were conducted with 10 participants in their homes.

**Results:**

Dimensions of poverty, particularly food and financial insecurity and insecure accommodation; unwanted pregnancy; and interpersonal conflict, particularly partner rejection, infidelity and general lack of support were reported as the causes of depression. Exacerbating factors included negative thoughts and social isolation. Respondents embraced the notion of task sharing, indicating that counselling provided by general health care providers either individually or in groups could be helpful.

**Conclusion:**

Counselling interventions drawing on techniques from cognitive behavioural therapy and problem solving therapy within a task sharing approach are recommended to build self-efficacy to address their material conditions and relationship problems in poorly resourced primary health care facilities in South Africa.

## Background

Maternal mental health is an internationally recognised public health concern [1-3] and the high burden of disease associated with postnatal depression is well documented in both high income countries (HICs) and low and middle income countries (LAMICs) [4,5]. Non-psychotic depression related to childbearing commonly affects between 10 to 15% of women worldwide [1]. In South Africa, current data suggest higher prevalence rates than these global estimates, with isolated studies on postnatal depression (PND) providing an estimated prevalence rate ranging from 16.4% in the township of Soweto to 39% in Khayelitsha, an informal settlement in the Western Cape [3,6,7]. In addition, the prevalence rate for antenatal depression, a predictor of PND, [8,9] has been found to be as high as 47% in a study in rural KwaZulu-Natal [8]. While maternal and child health is one of the top four public health priorities of the South African Department of Health, the emphasis is however, on decreasing physical morbidity and mortality [10].

However, in the face of a 12 month treatment gap of between 76% to 85% for people with severe mental disorders in low-income countries [11], and a75% treatment gap for common mental disorders in South Africa [12], the high burden of maternal depression poses a public health threat [13]. This is because PND can impact negatively on an infant’s socio-emotional and cognitive development as a result of maternal neglect, poor maternal responsiveness and impaired attachment relationship between mother and infant [5,14]. In turn, poor socio-emotional and cognitive development impedes long-term human capital growth that is essential for socio-economic development in LAMICs, having long ranging negative developmental, social and economic costs [5,15,16]. Untreated PND is also a human rights issue as it compromises codes of social justice for children who receive substandard maternal care as well as compromised quality of life for women with maternal depression [5].

Internationally, risk factors for maternal depression include dimensions of poverty such as low levels of education; poor housing and low income [17]; food insecurity and economic hardships [18]; stressful life events such as physical and emotional abuse; inadequate social support; history of depression; antenatal depression; low self-esteem; poor relationships with partners [3,15,19]; and unplanned and/or unwanted pregnancy [15]. In abusive relationships, income disparities and patriarchal systems that favour men render some women particularly vulnerable as they find it difficult to escape domestic violence due to their economic and or emotional ties to the abusive partner. Notwithstanding this literature, in order to inform culturally and contextually informed interventions, there is a need to understand how PND is experienced or understood within cultural contexts in LAMICs in order to inform culturally and contextually appropriate interventions. In addition, within resource constrained health care systems of LAMICs, there is a need to understand how best to deliver such interventions. Contextually appropriate and cost effective health care solutions for maternal depression need to be found [20]. Integration of mental health into primary health care and task sharing have been mooted as effective mechanisms to increase access to mental health care in LAMICs by the WHO (World Health Organization) and the South African Department of Health [21]. The objective of task sharing is to devolve tasks traditionally provided by specialist health workers to non-specialist health workers to provide increased access to quality health care at a lower cost [22]. Further, while many interventions for maternal depression in HICs favour home visits by nurses [18], in LAMICs resource constraints dictate that lay health workers are a more feasible option, with sufficient evidence of that they can be used effectively to carry out educational and psychosocial interventions for maternal care.

Against this backdrop, using Kleinman’s concept of explanatory models of illness [23] this study sought to explore the explanatory models of illness used by women diagnosed with PND, from low socio-economic backgrounds in South Africa. Specifically, the study seeks to understand the causes, course and treatment options available for their depressive symptoms. With regard to treatment options, in particular, the study sought to explore the acceptability of task sharing for the delivery of counselling interventions. This was with the view to informing the development of culturally and contextually appropriate maternal mental health services within the resource constraints of primary health care (PHC) in South Africa.

## Methods

### Study site

The study was conducted in a community health centre (CHC) in the Dr Kenneth Kaunda district in the North West Province. Economic activities in the district are predominantly mining and agricultural. The unemployment rate in the North West Province was 26.6% in the period the interviews were conducted, relative to 29.1% for South Africa as a whole [24]. Known as the “Platinum Province” with reference to its mineral wealth, mining is the mainstay of the North West Province, and typically attracts male employees. Some men have multiple families or partners, often both at a family home, and at the mining district. This is partly a historical product of the lack of facilities at mines to cater for families who are consequently left behind at home, sometimes in another province for lengthy periods [25].

The CHC is located in an urban township with a mixture of formal and informal dwellings. The facility provides both antenatal and postnatal services as well as a childbirth delivery service. HIV testing is routinely offered antenatally, and most women in the district give birth at health facilities and are attended by midwives. Home observations situate the context in which participants lived. Homes could be categorised as formal brick dwellings, basic Reconstruction and Development Programme (RDP) houses (low cost state subsidised two roomed homes with a kitchen and toilet), and informal shacks constructed with aluminium, cardboard and other materials. Access to electricity or inside ablutions is variable.

### Design

A focused ethnographic qualitative research approach using in-depth face-to-face interviews, follow-up interviews and observations of participants in the home environment was adopted. A qualitative study was deemed appropriate given the exploratory nature of the research [26]. Kleinman’s concept of explanatory models of illness [23] was used to guide the interviews. This framework typically seeks to understand the way people understand an illness in relation to the cause, course and treatment options for their symptoms. Focused ethnography is used to collect focussed information and assist with interpretations of cultures and systems.

### Recruitment and sample description

Fieldworkers explained the study to women attending services at the CHC postnatal waiting room. Women were randomly approached by a fieldworker in the waiting room and requested to participate. The criteria for participation in the study was women over the age of 18 not previously diagnosed with depression, who had given birth to a live infant who was at the time of the study aged between six weeks and twelve months old. Fifty-three women were screened over two months of which twenty women screened positive and were recruited. There were no refusals. A first stage screening instrument, the Edinburgh Postnatal Depression Scale (EPDS) was administered to participants as it is commonly used worldwide and has been validated for use in South Africa [27]. A cut-off score of 12 was used in this study as suitably predictive of PND [28,29]. A second diagnostic screen, the Structured Clinical Interview for DSM-IV (SCID-II).was used by two clinical psychologists to confirm diagnoses for major depressive disorder. Twenty participants, who met the criteria for a major depressive episode were recruited into the study over a period of 2 months and recruitment ceased once saturation of data was reached. Participants with a history of depression were excluded to ensure the focus on postnatal rather than general or chronic depression. Participants were compensated for their time with $5 (R50) supermarket vouchers. Participants’ demographic characteristics are detailed in Table 1.

**Table 1:** **Sample descriptives of women screened positive on EPDS and meeting criteria for major depressive episode**

**Demographic**	**N**	**Percentage**
Age		
Below 21	0	0
21 -30	10	50
31-40	9	45
41-59	1	5
Level of education		
No formal schooling	0	0
Primary	15	75
Secondary	2	10
Tertiary	3	15
Marital status		
Married	5	25
Cohabiting	10	50
Single	5	25
Employment status		
Employed	4	20
Unemployed	16	80
Sources of income		
Salary only	8	40
Salaries and social grants	5	25
Pension and other social grants	2	10
Social Grants only	2	10
Social grants and other sources such as families	1	5
No minimum income (rely on families and other sources)	2	10

### Data collection

With the participants’ consent, semi-structured, audio-recorded interviews were conducted in Setswana by two clinical psychologists to explore participants’ experiences and explanatory models of postnatal depression, their symptoms and coping strategies. Ten follow-up interviews were conducted with available participants from the first wave of interviewees two months later in their homes. The objective of the follow-up interviews was for participants to confirm earlier findings, to gain a better understanding of participants’ home environments and social contexts, and observe their interactions with family members and infants. The analysis of the first and second set of interviews was combined.

### Data analysis

The interviews were translated and transcribed into English with back-translation checks being applied by an independent English/Setswana speaker. Thematic analysis was both theory and data driven. Interviews were guided by specific research questions but the participants were encouraged to respond to the questions and to add any related information that they wanted to share. This approach was used to ensure that the essential data that was required to assist with developing the intervention would be collected and that any other data that was collected not specifically related to the research questions but related to the interview would add richness and depth to the data and would be useful to guide the intervention. The data was thus coded using apriori themes based on the interview questions as well as emergent themes with the help of Nvivo qualitative software for data analysis. The first and second authors independently generated themes and coded the data. The generated themes and coded data were compared and consensus reached on the final coded data.

### Ethical considerations

Ethical approval was obtained from the Humanities and Social Sciences Research Ethics Committee at the University of KwaZulu-Natal (ethical clearance number HSS/0880/011), and the Policy, Planning, Research, Monitoring and Evaluation division based at the Department of Health in the North West Province. The information and consent sheets were verbally explained to potential participants who were then given the opportunity to read the information sheets and ask questions confidentially. Participants were recruited following informed consent in their preferred language i.e. either Setswana or English. Participants who met the criteria for a major depressive episode and/or suicidal ideation were referred for further assessment and treatment using existing referral pathways.

The methodological guideline for qualitative research reporting (RATS) [30] was adhered to in the reporting of the qualitative study in this paper.

## Results

### Symptoms of depression

The most common symptoms described by participants included wanting to cry; not taking care of one’s appearance and feeling the need to be alone; physical symptoms such as headaches and neck tension, sleeplessness and loss of appetite; feeling impatient, irritable and angry; and feeling down were also mentioned.

*“I would feel angry for no reason. I would also feel like crying; feeling as if I am going through deep pain; feeling like wailing” (Case 11).*

And

*“One finds it difficult to sleep; doesn’t have appetite; is too forgetful; is impatient, things like that” (Case 12).*

### Expected course and resolution of the depression

Participants indicated that they did expect to get better after receiving help.

*“I would not lose my mind anymore; I would become calm after receiving help” (Case 9).*

### Causes of depression

Participants generally described their experiences of depression as being the result of adverse circumstances and life events that caused feelings of pain and sadness. While some participants understood the term “depression” and were able to verbalise and relate their symptoms as depression, others were not aware of what the term “depression” meant. Further probing with these participants regarding the cause of their symptoms elicited similar responses and themes that were provided by those participants who were aware of the term and meaning of depression.

Key sub-themes included poverty and dependence; interpersonal conflicts, particularly with partners and families; unwanted pregnancy and social isolation.

#### Poverty and dependence

Poverty through associated stressors emerged as a pervasive causal factor of depression, in particular food, housing and financial insecurities as reflected in the quote below.

*“If I could get a job and also continue with my studies. If I could make sure my children, mother and my siblings live a better life; where there is nothing to bring me pain; when there is no day in which we go to bed without having eaten. That’s when I may feel my life has really improved.” (Case 5).*

In South Africa, there is significant reliance on the child support grant (CSG), an initiative of the State’s Social Assistance Programme to combat abject poverty and hunger, and whose beneficiaries are poor children under the age of sixteen. Recipients are usually mothers or caregivers [31]. Sometimes the CSG was used to supplement food purchases for the household, and there was also reliance on other family members’ social grants such as state old age pensions. The CSG at the time of the interviews was $29 (R290) per child, per month. In response to being asked who buys the food at home, one participant responded.

*“It’s my mother. She is the one who used to buy 50 kg of maize meal after getting her pension money. So this month that has been difficult for her to do as she wasn’t able to get the usual pension money. So I managed to buy some maize meal using the grant money I get for my children.” (Case 5).*

And

*“I earn a living from the children’s welfare grants.” (Case 2).*

Poverty renders mothers dependent on state support and/or families. These supports are often inadequate or withheld, resulting in feelings of despair and helplessness. Combined with a lack of education and formal marketable job skills to enable them to generate income, participants became trapped in a negative cycle of dependence and poverty. The situation was exacerbated in some cases where, because of poverty, the mother was rendered dependent on an abusive partner and unable to extract herself from the abusive relationship due to financial dependency. This was the case for a significant proportion of respondents (seven).

*“You try to leave your parents’ place to go and stay with your partner, and when he hurts you, you would not know where to go from there; you feel like there is nothing that you can do. And you end up continuing to stay with this person as you have no other alternative” (Case 14).*

### Partner conflicts

Serious conflict with partners emerged as triggers of depression including rejection by the partner, lack of material and/or emotional support, disinterest in the child and denial of paternity; physical abuse; and infidelity.

#### Rejection/lack of support by partner

A number of participants (nine) reported that although their partners acknowledged paternity, they did not provide financially for the children. Nor do they assist with parenting or provide emotional support to the mothers.

*“Yes, one thing that pains me the most is that the father of my child never comes to check on us. That’s what I am always worried about. He does make phone calls saying he would come over for the weekend, but he never does that. I would tell him about some of the child’ needs that are lacking and he will ask me why I can’t provide for the child’s needs with Zuma’s money; he will be referring to the grant money […] He never denied that he fathered my child. Ever since the child was born he has never done anything for him; I mean nothing at all. Not even to buy him some clothes. Mind you the child was born in July when it’s usually very cold. I have been struggling trying to make ends meet using only the grant money I get for my first child. His child is a year old and knows how to walk but, he has never seen him”. (Case 2).*

#### Infidelity

Six participants also reported that their partners were unfaithful to them, reporting this as a major stressor.

***“****When the baby was two months old, I hastened to come back as I heard that he was staying with a girlfriend at our house and she was using my things.” (Case 18).*

And

*“His aunt asked him why he never told them about the pregnancy, why it took him so long and why he wanted me to commit abortion. He told them that he has a girlfriend in Lesotho whom he has promised to marry, thus he is afraid of disappointing her” (Case 1).*

Associated with infidelity was the stress of the spectre of HIV and other sexually transmitted diseases. There seemed to be little discussion or negotiation or an inability to negotiate around issues of protection and prevention as evidenced below.

*“When I was eight months pregnant I came here to test for HIV and I was very concerned that I could be infected as he has been unfaithful” (Case 5).*

And

*“He continues to go and collect other people’s infections and bring them to (me)” (Case 13*).

#### Intimate partner violence

Some participants (six) also reported experiences of intimate partner violence as triggers of depression.

*“…When he comes home from visiting his friends or from drinking alcohol; when he arrives drunk like that and finds us sleeping, he has the tendency to be violent; he will take objects and hit me with them while hurting me in the process… So I always think about this thing; it really affects me mentally.” (Case 10).*

An added economic burden associated with material support was the expectation of financial support from the male partner’s family members sometimes at the expense of the woman and her child.

*“I always get to hear what they say about me in my absence. Like they say I use up their brother’s money. My mother in-law would say how much trouble she went through while raising her son” (Case 20).*

### Unwanted pregnancy

Having had an unwanted pregnancy was itself a significant stressor and reported trigger of depression for varied reasons. Women who were unhappy in their intimate relationships viewed an unwanted pregnancy as a complication that bound them unwillingly to the relationship. This is articulated in the quote below.

*“And I wondered as to what I could do. I could see that being pregnant with this child meant I could no longer leave the marriage unit. Committing abortion was no option as I knew I was HIV positive, or else CD4 count would drop down and I will start to become sick which means I would be taking a risk”. (Case 13).*

Reasons for unwanted pregnancy included unplanned pregnancy; inability to support the child and lack of support from the partner. In response to being asked how they felt when they realised that they were pregnant, responses included

*“Imagine a situation whereby you are pregnant and you tell this person who impregnated you about it. After telling him, he starts behaving in a way that shows he is not with you in the situation. You start to wonder, ‘am I going to handle all this by myself;’ you don’t see the importance of keeping the baby. I am unemployed, so who is going to help me raise the child?” (Case 2).*

### Exacerbating factors

#### Lack of supportive family relationships

Lack of support from parents and/or family was reported by 14 participants and contributed significantly to the burden of depression. The respondent quoted elsewhere above who experienced intimate partner violence further elaborates.

*“…When I report this matter to my family they would tell me that he is my husband and he has paid the dowry therefore there is nothing that they could do. I have told myself that I am all alone in this matter. There is no one who cares about me.” (Case 10).*

Lack of support from the partner’s family was also reported by 7 participants as particularly difficult to deal with and contributed to their stress.

*“The in-laws didn’t accept the baby. The reason for the way they act is because they say that before coming to give birth my family should have written them a letter letting them know about the pregnancy, and another letter should have been written to them after the child had been born. That’s what is demanded by their culture. So, all that they are doing (not acknowledging the child) is because now it was as if they were never involved at first”. (Case 2).*

#### Social withdrawal and unhealthy thinking

Social withdrawal and unhealthy thinking which are symptoms of depression also functioned to exacerbate depression, trapping people in the negative cycle of depression and/or social withdrawal

*“There are times when I am not able to look after myself properly. Like there are times whereby I would shut myself in the house and ensure I don’t go anywhere” (Case 11).*

And

“*So in regards to people out there may be if a friend comes and says ‘I have just come to see you’; you don’t really have the interest to chat with the other person or even face the person” (Case 10).*

A disturbing number of participants (seven) reported suicidal ideation or suicide attempts.

*“I overdosed some pills; sometimes I would drink jik” (Case 1).*

Although voluntary termination of pregnancy (TOP) is legal in South Africa and available to public health care users, there seemed to be a lack of education among some women about these services and when to use them as some of the respondents indicated that they were informed that their pregnancies were too advanced to carry out a TOP. This participant attempted suicide as a result of her unwanted pregnancy. She felt that suicide was the only option to terminate her pregnancy.

*“That’s the time when I tried to kill myself so that I could kill the child”. (Case 15).*

Excessive worrying, blame and distorted thinking in the form of repetitive negative intrusive thoughts was reported by eleven respondents. These harmful thought patterns influenced mood negatively. Unhealthy thought patterns maintain and perpetuate harmful thoughts and divert attention from making efforts to achieve their aspirations.

*“Is there anything that would make me never think that I am a failure in life? I want to know because I am always miserable and this is due to the fact that I always blame myself for having children?” (Case 5).*

*“I get angry at myself for bringing children on this earth only to come and suffer just because of me.” (Case 10).*

And

*“I blamed his father because I had told him that I wasn’t yet ready for us to go and have sex. If it wasn’t because of him I wouldn’t be having a child and now the child has been born but he is now suffering. He (partner) has brought me problems” (Case 2).*

#### Negative behaviour

Other negative symptoms and feelings reported included acting out their frustrations by hitting their children, neglecting their hygiene needs and scolding them disproportionately, including their new-borns. They reported irritability and anger when interacting with their children.

*“Yes. I do beat them up. When I am angry I would also start to shout at them. Like I would yell and force them to sleep. When they don’t sleep and start to cry I will slap them.” (Case 4).*

And

*“Sometimes when I have to make her take a bath or wash her nappies I would feel too lazy to do anything and I would leave everything as it is”. (Case 5).*

Similarly, interpersonal violence is destructive to relationships and inappropriate as a solution to problems. Unhealthy behaviour such as the incident quoted below is indicative of extreme frustration and not having the tools to deal with such levels of emotion.

*“So I was very furious; I took anything I could reach and tried to throw at him. I ended up getting a knife and stabbed him with it on the hand.” (Case 20).*

### Treatment options for postnatal depression

None of the participants mentioned seeking any assistance for their problems either from religious, social or medical structures while one woman indicated that she did think about consulting a doctor for her depression but was afraid that her partner would not allow this. The absence of help-seeking could possibly be attributed to the overwhelmingly interpersonal and economic nature of the problems as the quote below illustrates.

“*Depression is stress. At times your distress is elicited by your partner. At times your partner would not give you even a cent; nothing at all and that’s when you become depressed.” (Case 10).*

The problem related above and the attribution of the cause of depression suggests that in such cases medical intervention is not considered appropriate to address the problem, and the perceived stigma associated with articulating these issues might prevent help-seeking from religious and social structures.

Task sharing in terms of devolving counselling duties from specialists to non-specialist health workers was acceptable for most respondents with eighteen respondents indicating acceptance of counselling from community health workers and sixteen from nurses. Anonymity rather than expertise in mental health care was the principal criteria for some women to accept counselling from nurses and CHWs.

*“I need someone that I can talk to, especially someone who doesn’t know me and that I do not know; someone who is not judgmental”. (Case 2).*

Attending a support group received mixed support. Some respondents expressed reluctance to attend support groups. Five respondents suggested group-based support as a useful intervention given that women would be able to share their problems with people in similar situations.

*“When we have met as a group we would be able to discuss the things that cause us to suffer from depression and also find out how we can help each other”. (Case 9).*

In response to being asked if nurses would be suitable to lead support groups, this participant responded

*“I wonder. I think they can try to offer some little help but, I think someone who has had an experience of suffering from depression should be the leader of a support group” (Case 9).*

This particular quote shows a desire for empathy, understanding and personal experience related to depression as being desired qualities for a group leader. There is no expectation that the group leader would have a mental health or medical background.

Some women expressed a preference for treatment during home visits

*“Since it may not always be possible (for people to attend counselling sessions at the clinic), I was thinking may be door-to-door visits to people at their home could be done.” (Case 12).*

Access was also a consideration for attending groups:

*“… In a site which I would only have to walk in order to go and attend the meeting because if it was in town, at times I wouldn’t have money to go there”. (Case 14).*

## Discussion

Poverty, unwanted pregnancy and interpersonal conflict including partner rejection and associated lack of material and social support as well as conflicts including domestic violence, emerged as significant stressors that triggered depression in common with local and international literature [3,8,32]. These findings suggest that the reasons for maternal depression in women from low socio-economic backgrounds in the study site are not different to that of other maternally depressed women globally. Exacerbating factors included unhealthy thinking in the form of negative intrusive thoughts as well as social isolation which are typical symptoms of depression that maintain the depressive cycle. Suicidal ideation and suicide attempts revealed the depths of hopelessness that some of the women experienced.

Pregnancy and childbirth are particularly vulnerable periods for a woman, particularly first pregnancies as these involve changes to her body image, her identity as a woman, concepts of motherhood, changed intimate relationship variables and responsibility for a helpless infant. Combined with partner relationship problems and/or socio-economic difficulties, these contribute to many women’s experiences of PND. The impact of childbirth is also in many contexts culturally determined [33], and when it is not validated by the traditionally expected support structures such as families, it can be extremely distressing .Postnatal depression is especially evident in the context of unwanted pregnancies; rejection of the pregnancy by the partner, and lack of support for the woman and child from the partner’s family. This needs to be understood within African culture where childbearing in women is highly valued.

Although poverty per se does not inevitably result in depression, experiences of living in poverty stricken conditions interrelate in a vicious cycle which impact on mental health status, childcare and self-care [5]. Results of this study indicated that food, financial and housing insecurity rendered some women economically dependent on their male partners, which made them vulnerable to intimate partner violence and abuse. Low educational status combined with a depressed economic climate and high unemployment rates promotes economic dependence and contributes to a sense of hopelessness. A recurring refrain was the desire to find employment. For poor women with low educational backgrounds, few or no job skills and harsh economic conditions further reduced hopes of securing employment. In 2010/2011, increased social assistance, translated to an increase of 0.3 percent of the gross domestic product [31]. Social welfare meant to be a buffer against poverty is increasingly the sole source of income in poor households.

A high rate of circular male migration related to employment on the mines [25] and the common practice of polygamy or having multiple concurrent partners may contribute to the problem of infidelity, abandonment and low levels of support by male partners, which emerged as a prominent theme. While the African traditional custom of affiliation to ancestral lands encourages the practice of having a family based at the ancestral village/home, economic factors dictate work-seeking in more urban and economically advantaged areas. Long absences and distance to the workplace mean that spouses experience long periods away from each other, with men often taking up a second relationship at their workplace.

Results of this study suggests that the socio-economic/ family context causes additional economic burden on the male partner as he may be expected to support his extended family with limited resources possibly skewing the economic support in the family’s favour and depriving the partner and child of economic support or causing friction between the parties competing for financial support. This type of economic burden is not unusual as in South Africa, the male breadwinner is often the sole economic support for multiple households and one male can support up to 10 family members [34]. Patriarchal culture where men hold economic, cultural and religious advantages over women and engenders female dependence on men for their economic and social security [30] also emerged as playing a role in PND in this study. Six women out of a total of twenty one revealed that they had experienced abuse by their partners. Women all over the world experience these issues [35,36], and although contexts differ according to regional beliefs and practices, interpersonal violence (IPV) is a reality in both high and low income settings [37]. In another study in South Africa 31% of women indicated that they had experienced some form of IPV in their most recent relationship [35]. IPV renders women vulnerable to physical nonfatal and fatal health outcomes including suicide [36] and intimate partner femicide [37]. The destructive physical and psychological effects of IPV include greater risk of substance abuse and increased health care seeking [35], and contribute significantly to the burden of disease in South Africa [38].

The lack of commitment from male partners who demonstrate little interest in fulfilling financial and parental obligations or fidelity in their intimate relationships compounds the socio-economic difficulties and creates fragmented families who are related biologically but who share few or no familial ties. Weak paternal bonds breed dysfunctional relationships in families and society as a whole. Infidelity on the part of the male partner was a common occurrence, particularly when the woman was pregnant or in the confinement period postpartum. There was also little mention of responsibility for contraception or concern for safe sex practices. In some cases any concerns about possible HIV infection that the women voiced appeared to be after they realised that their partners had been unfaithful. A disturbing aspect is despite the high rates of HIV infection in South Africa, there seemed to be little negotiation of safe sex in intimate partnerships in the responses from this study. Additionally, despite the legalisation of termination of pregnancy and concerted efforts to promote contraceptive use, rates of unwanted pregnancy are unacceptably high in South Africa [39], and require further investigation.

An interesting paradox was the concurrent self-blame and blame-shifting for participants’ unhappiness. Many women either blamed themselves for making unwise choices, or blamed their partners or partners’ families for their unhappiness and feelings of depression. The assigning of blame caused depressive feelings, unhappiness and repeated intrusive thoughts related to past hurts. The inability to attempt to break destructive behaviour patterns might suggest unhealthy thinking and faulty cognitive behavioural patterns which is disempowering.

The results of our study indicate that individual’s personal experiences of social and economic factors may precipitate or exacerbate depression. The complexity of addressing symptomology of depression together with predominantly socio-economic causes suggests the need for multiple interventions at various levels of the problem. While social, household and economic factors often precipitate and exacerbate symptoms of depression, it is important to attend to the most proximal causes before attempting to deal with more distal-related events that are often out of the control of the individual. Alleviating the symptoms of depression through focusing on re-building a sense of agency through promoting self-efficacy and problem-solving approaches within a supportive group environment has been noted to help individual’s cope with their depressive symptomatology [40,41].

The participants also voiced strong support for either individual or group-based counselling services and for devolving these tasks to non-specialist health workers. This type of intervention could be made available both at facility and community levels, depending on the cadre of health worker assigned. Encouragingly, participants who indicated a preference for groups expressed wanting the social support that would emanate from a group and being with others who would empathise with their feelings. This approach is encouraged by Dennis and Hodnett [42] in their review of psychosocial interventions for PND who suggest further research should focus on self- help groups (not led by a health professional) to evaluate lay support models for mild to moderately depressed women. In addition, groups would have the added benefit of allocating one health worker to a number of participants at the same time which would be more cost effective in terms of time spent on a single intervention, travel time and travel costs to the health care system and to the community. The social network of the group could also assist participants to build social capital and form social and economic networks.

At the community level prevention and mental health promotion interventions require a multi-sectorial response from educational, social development and health agencies. Concerted efforts need to be made to promote the role of men as caring, responsible partners and fathers. This requires sustained and targeted campaigns to address issues including domestic violence, the negative effects of multiple concurrent sexual partnerships, shared responsibility for contraception and shared responsibility for children. These community based interventions should be on-going at schools, places of socialisation, places of worship, health facilities and other public forums. In addition, microfinance and income generating opportunities for women need expansion. Promising results from the IMAGE study [43] in South Africa which combined a microfinance program with training on HIV infection, gender norms and domestic violence indicate that economic and social empowerment of women can contribute to a reduction in IPV. This synergistic combination of economic empowerment with a public health intervention suggested multiple benefits for the women, their family members and the community.

### Limitations

This study is limited to a small number of participants from a specific region. Within the ambit of the study i.e. PHC users, participants are not representative of all PHC users. There is a need for further studies in PND in other PHC sites in South Africa in order to adapt and standardise brief psychosocial interventions for women with PND. This approach will assist with sustainability of training and supervision mechanisms and continuity of selected psychosocial interventions.

## Conclusion

The high prevalence rates for maternal depression in South Africa warrant far more attention than is currently provided at PHC level given that the majority of the population does not have private health insurance and will use PHC services. Notwithstanding the small sample, this study provided some valuable insights into the issues that mothers, particularly in low socio-economic groups face. As evidenced in this study, interventions for maternal depression require a multilevel approach. At the facility level, routine screening for depression should be made at all ante-natal and post-natal clinic visits up to a year after giving birth. In addition to availability of anti-depressant medication for severe depression, such screening should be accompanied by referral pathways for psychological interventions that have been shown to be effective within a task shifting approach [44] to promote social support and empowerment of women to better manage the psychosocial stressors that have been found to trigger depression. To this end, evidenced-based brief targeted psychosocial interventions may be applicable to address the triggers and symptoms of depression. These may include techniques such as Cognitive Behavioural Therapy (CBT) to reduce symptoms of depression and co-morbid anxiety, and Problem Solving Therapy (PST) techniques to help develop problem solving skills to address social and economic issues [45]. These modalities can assist with symptom management and self-efficacy, but should ideally be accompanied by inter-sectorial actions to promote gender equity and socio-economic development.

## Abbreviations

LAMICs: Low and middle-income countries
PND: Postnatal depression
HICs: High income country
WHO: World Health Organization
CHWs: Community health worker/s
PHC: Primary health care
CHC: Community Health Centre
RDP: Reconstruction and Development Programme
EPDS: Edinburgh postnatal depression scale
SCID: Structured Clinical Interview for DSM-IV (SCID-II)
CSG: Child support grant
IPV: Intimate partner violence
